# Multiple jejunal gastrointestinal stromal tumors and Neurofibromatosis type 1: A rare association

**DOI:** 10.1016/j.ijscr.2021.106178

**Published:** 2021-07-07

**Authors:** Aakash Mishra, Sandesh Gyawali, Sanjeev Kharel, Aman Mishra, Nibesh Pathak, Nirajan Subedi, Prabin Gaire

**Affiliations:** a Kathmandu Medical College Teaching Hospital, Kathmandu, Nepal; b Department of General Surgery, National Academy of Medical Sciences, Bir Hospital, Kathmandu, Nepal; c Maharajgunj Medical Campus, Institute of Medicine, Maharajgunj, Kathmandu, Nepal; d Department of GI and General Surgery, Maharajgunj Medical Campus, Institute of Medicine, Kathmandu, Nepal; e Department of Pathology, Maharajgunj Medical Campus, Institute of Medicine, Kathmandu, Nepal

**Keywords:** Neurofibromatosis type 1, Gastrointestinal stromal tumor, NF1-associated GIST, Association, Multiple tumors

## Abstract

**Introduction and importance:**

The association between gastrointestinal stromal tumor (GIST), mesenchymal tumor arising from the interstitial cells of cajal and Neurofibromatosis type 1 (NF1), an autosomal dominant disease has been reported in the literature. GIST in NF1 patients are multiple and located in the small intestine. Tumorigenesis in NF1 associated GIST is different to that of sporadic GIST and hence the treatment. Here we report a rare case of an NF1 patient with multiple jejunal GISTs.

**Case presentation:**

We here present a rare case of a 57-year-old male diagnosed with NF1 30 years back, presented in our emergency department with complaints of black, tarry stools later diagnosed to have multiple GIST in jejunum. Contrast enhanced computed tomography (CECT) of the abdomen showed a large 10.1 × 7.33 × 6.2 cm heterogeneous, exophytic, solid mass with cystic areas originating from the jejunum. The microscopic examination of the specimen showed spindle shaped tumor cells while immunohistochemistry showed CD117 (c-KIT) and DOG-1 positivity. The primary treatment was complete surgical excision of the tumor.

**Clinical discussion:**

The incidence of GISTs in NF1 patient is around 6–7%; however, concomitant presence of multiple GISTs is rare. CECT of abdomen along with histopathological and immunohistochemistry studies are diagnostic. The management of GIST includes surgical and adjuvant therapy methods based on the tumorigenesis and recurrent risk stratification.

**Conclusion:**

Early clinical suspicion and imaging aids in early detection of the tumor in patients with NF1 presenting with gastrointestinal symptoms. Postoperatively, screening for recurrence with radiology is of utmost importance.

## Highlights

• An association between Neurofibromatosis 1 (NF1) and gastrointestinal stromal tumors (GIST) has been reported in literature.
• Clinical suspicion for GISTs should be made in middle-aged or older NF1 patients presenting with gastrointestinal symptoms.
• CECT of the abdomen along with histopathological and immunohistochemistry studies are diagnostic.
• The treatment often mandates surgical resection of the tumor and the prognosis of NF1-associated GIST patients is good.

## 1 Introduction

Neurofibromatosis type 1 (NF1) has an incidence of 1 in 4000 in the general population and is a common autosomal dominant genetic disease with varying clinico-pathological features and an uncertain disease course [1], [2]. Mutation in NF1 gene, which encodes neurofibromin, causes loss of its function and results in Ras activation, promoting the tumor formation [3]. GIST is a rare mesenchymal tumor occurring in the gastrointestinal (GI) tract with incidence in less than 1% of all gastrointestinal tumors. A number of published literatures have shown an association of GIST with NF1 [2], [3], [4], [5], [6]. An alternative molecular pathogenesis has been reported among cases of NF1-associated GIST suggesting differences within the general population. This information is very important for therapeutic implications [7].

This work has been reported in line with the SCARE criteria [8].

Herein, we present an unusual case of multiple GISTs originating from the jejunum in a patient with a 30 years long diagnosis of Neurofibromatosis type 1.

## 2 Case report

A 57 years male, a known case of Neurofibromatosis type 1 diagnosed 30 years ago, presented to our emergency department with the complaints of black, tarry, foul-smelling stools for three days and multiple episodes of acute-onset, non-projectile, non-bilious, non-blood stained vomiting. The patient was pale on examination. A hard, non-tender mass in the umbilical region could be palpated. Diffuse cutaneous lesions of neurofibromatosis were also present. The examination of all other systems was grossly intact.

Complete hemogram of the patient revealed severe iron deficiency anaemia with thrombocytosis. Routine blood biochemistry parameters of the patient were within normal limits.

**Fig. 1:**
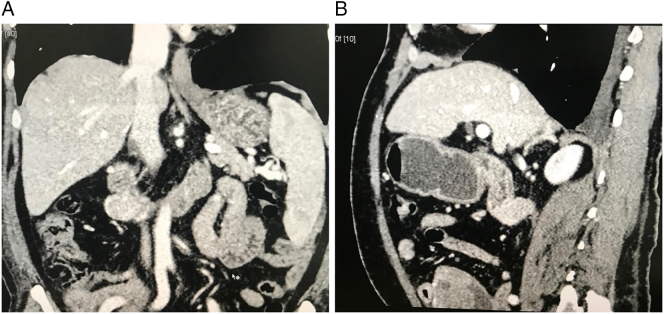
(A and B): Coronal and sagittal plain showing a large 10.1 × 7.33 × 6.2 cm heterogeneously enhancing, exophytic, complex, solid and cystic mass arising from jejunum.

Abdominopelvic ultrasonography (USG) revealed an approximately 5 × 5.5 cm, well defined, circular, predominantly hypoechoic area and an echogenic area nearly 2.5 × 2.3 cm with no vascularity in the left paraumbilical region. Contrast enhanced computed tomography (CECT) of the abdomen (Fig. 1A,B) showed a large 10.1 × 7.33 × 6.2 cm heterogeneous, exophytic, solid mass with cystic areas originating from the jejunum and supplied by the superior mesenteric artery. These findings were suggestive of gastrointestinal stromal tumor.

**Fig. 2:**
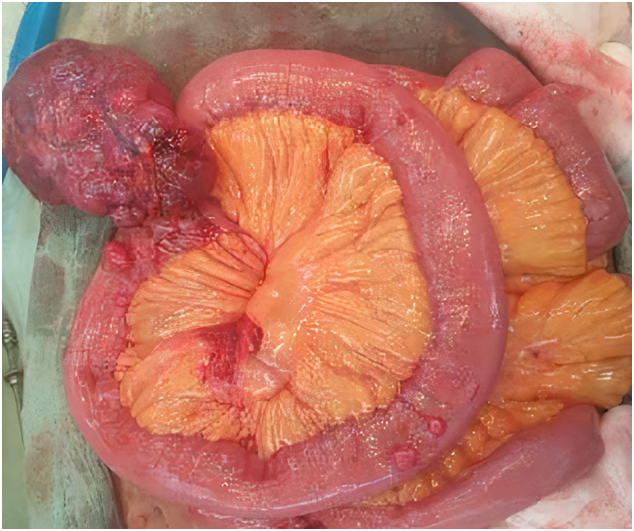
Intra-op findings: 6–7 cm oval mass located in the antimesenteric border of proximal jejunum about 50 cm distal to the duodeno-jejunal flexure.

An exploratory laparotomy was done. Approximately 6-7 cm oval mass located in the antimesenteric border of proximal jejunum about 50 cm distal to the duodeno-jejunal flexure was seen (Fig. 2). The mass along with the jejunal segment approximately 10 cm proximal and 5 cm distal to the mass was resected and a jejuno-jejunal anastomosis was made.

The postoperative hospital stay was uneventful and patient was discharged on 7th post-operative day. On follow up after eight weeks, he had no new complains and physical examination was insignificant. CECT revealed no new growth and advised to follow-up every three months for one year.

**Fig. 3:**
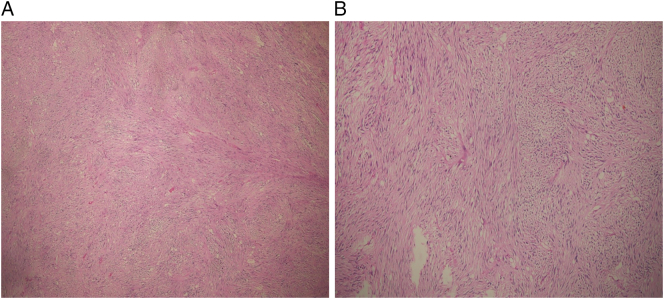
(A and B): A. H&E, ×40 magnification. B. H&E, ×200 magnification. Showing spindle shaped tumor cells arranged in fascicles and bundles occupying the submucosa and muscular layer.

**Fig. 4:**
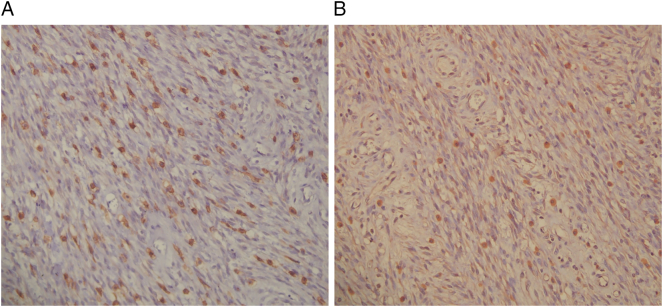
(A and B): A: Immunohistochemistry of tumor cells (200×) showing focal weak membranous immunoreactivity for CD117. B: Immunohistochemistry of tumor cells (200×) showing focal weak membranous immunoreactivity for DOG1.

Macroscopic examination of the resected specimen showed seven submucosal small to large nodular masses extending up to the serosa wall. The size of the tumors ranged from 0.5 cm, being the smallest up to 10.5 cm, being the largest. The largest mass was in continuity to the mucosal surface which was ulcerated, which explains the few episodes of black to dark red tarry stool he passed. On microscopic examination of the specimen, spindle shaped tumor cells arranged in fascicles and bundles occupying the submucosa and muscularis propria were seen (Fig. 3A,B), suggestive of a spindle cell neoplasm most likely Gastrointestinal stromal tumor. The tumor had a histological grade G1 with 1/50 hpf (high power field) mitotic figures with no lymphatic, vascular and perineural invasions. TNM staging (according to AJCC/CAP protocol 2020) was at pT4 stage. The diagnosis was confirmed by immunohistochemistry positive for CD117 (c-KIT) and DOG-1 (Fig. 4A,B). The patient falls under high risk category according to Joensuu criteria [9], [10].

Based on the clinical features, radiological and histopathological findings, a diagnosis of multifocal jejunal gastrointestinal stromal tumor (GIST), spindle cell type was made.

## 3 Discussion

GIST is an uncommon tumor of mesenchymal origin and even rare when multiple in number in a single patient [9], [11]. The mechanism of tumorigenesis in case of NF1 associated GIST is different from that of sporadic GIST, hence the disease presentation and prognosis are different. In our case, a rare presentation of seven such tumors, diagnosed as multiple GISTs was seen.

There is a strong association between NF1 and GIST as indicated in various case reports and case series [5], [12]. NF1-associated GIST presents at a younger age (median age: 49 years) while only sporadic duodenal or jejunoileal GISTs are seen at later age (median age: 56 years). Female predominance is found in most of the cases [13]. In contrast, here we present a case of a 57-year male with NF1 and multiple GIST (seven in number). NF1-associated GIST is due to an increased signal transduction via the MAP-kinase pathway resulting from somatic inactivation of the wild-type NF1 allele [14]. Tumors are strongly positive for KIT by immunohistochemistry but mutations in KIT or PDGFRA (Platelet Derived Growth Factor Receptor Alpha) genes are rare among NF1 associated GISTs, contrast to the sporadic cases of GIST where somatic mutations in KIT or PDGFRA genes play a major pathogenesis [9], [11], [15]. The characteristics like higher degree of spindle cell morphology, CD34 expression along with tendency to be multifocal occurring in the small bowel are helpful to differentiate NF1 associated GIST and sporadic GIST [6,14]. There is an increased risk of developing GIST in NF1 patients than the general population. The incidence of GISTs in NF1 patient is around 6–7% not including undiagnosed and commonly missed asymptomatic and small GISTs [16].

NF1-associated GIST is difficult to diagnose because of unusual location of tumor in small intestine. We sent the patient for CECT and rushed the patient to the operating room owing to ongoing blood loss. However, newer diagnostic tools like magnetic resonance imaging (MRI), or positron emission tomography, CT or ultrasound-guided needle biopsies or laparoscopy are helpful for diagnosis in patients presenting to the out-patient department [17], [18].

According to the extent of disease, the management of GIST includes surgical and adjuvant therapy methods. The cornerstone treatment is surgical for primary resectable disease along with chemotherapy as adjuvant therapy based on the recurrence risk stratification.

In our case, the patient presented to the emergency department and an emergency laparotomy with surgical resection of the mass was performed. Jejunal segment approximately 10 cm proximal and 5 cm distal to the mass was also resected considering GIST or adenocarcinoma of small intestine as our differential diagnosis, since confirmed histopathological examination was not feasible in the emergency setting.

While for wild-type cases of NF1, adjuvant therapy with imatinib is considered on a case basis [14], [19]. But recent studies have shown imatinib ineffective or not recommended against NF1-associated GIST [20]. This may be related to the rarity of c-KIT mutations among NF1 associated GISTs. In contrast, studies have shown use of sunitinib to be helpful in NF1-associated GIST [21]. Owing to the size of the tumor, our case fell under high risk of Joenssu criteria. Adjuvant therapy with sunitinib or imatinib along with surgery would have been an option in this case but due to the unavailability of sunitinib in our country and ineffectiveness of imatinib in the GIST associated with NF1, only surgical resection was done. The prognosis of patients (overall survival and recurrence free survival) of NF1 associated GIST patients is good.

## 4 Conclusion

There must be a high index of suspicion in middle aged or older NF1 patients presenting with gastrointestinal symptoms for GISTs. Prompt evaluation should be done by radiological studies like abdominal CT or MRI scan for early management and effective treatment to reduce fatal outcomes.

## Ethical approval

Not required.

## Funding

None.

## CRediT authorship contribution statement

AM: Study concept, Manuscript preparation

SG: Manuscript preparation

SK: Manuscript preparation

AM: Manuscript preparation, Critical review of study

NP: Surgical management and care of patient, Manuscript preparation

NS: Surgical management and care of patient, Supervision of study

PG: Histopathological studies

All authors were involved in reviewing the final draft of the manuscript.

All authors have made a significant contribution to preparing the case report.

## Guarantor

Aakash Mishra accepts full responsibility for the work and/or the conduct of the study, had access to the data, and controls the decision to publish.

## Registration of research studies

Not applicable.

## Consent for publication

Written informed consent was obtained from the patient for publication of this case report and accompanying images. A copy of the written consent is available for review by the Editor-in-Chief of this journal on request.

## Note

No patient and author details are included in the figure.

## Provenance and peer review

Not commissioned, externally peer-reviewed.

## Declaration of competing interest

Nothing to state.
